# Recent Insights into *Clostridium perfringens* Beta-Toxin

**DOI:** 10.3390/toxins7020396

**Published:** 2015-02-03

**Authors:** Masahiro Nagahama, Sadayuki Ochi, Masataka Oda, Kazuaki Miyamoto, Masaya Takehara, Keiko Kobayashi

**Affiliations:** 1 Department of Microbiology, Faculty of Pharmaceutical Sciences, Tokushima Bunri University, Yamashiro-cho 770-8514, Tokushima, Japan; E-Mails: cfcdv305@jtw.zaq.ne.jp (K.M.); mtakehara@ph.bunri-u.ac.jp (M.T.); kobakei@ph.bunri-u.ac.jp (K.K.); 2 Department of Microbiology, Fujita Health University School of Medicine, Toyoake 470-1192, Aichi, Japan; E-Mail: ochi@fujita-hu.ac.jp; 3 Division of Microbiology and Infectious Diseases, Niigata University Graduate School of Medical and Dental Sciences, Gakkocho-dori, Chuo-ku 951-8514, Niigata, Japan; E-Mail: masataka@dent.niigata-u.ac.jp

**Keywords:** *Clostridium perfringens*, beta-toxin, necrotizing enterocolitis, pore-forming toxin

## Abstract

*Clostridium perfringens* beta-toxin is a key mediator of necrotizing enterocolitis and enterotoxemia. It is a pore-forming toxin (PFT) that exerts cytotoxic effect. Experimental investigation using piglet and rabbit intestinal loop models and a mouse infection model apparently showed that beta-toxin is the important pathogenic factor of the organisms. The toxin caused the swelling and disruption of HL-60 cells and formed a functional pore in the lipid raft microdomains of sensitive cells. These findings represent significant progress in the characterization of the toxin with knowledge on its biological features, mechanism of action and structure-function having been accumulated. Our aims here are to review the current progresses in our comprehension of the virulence of *C. perfringens* type C and the character, biological feature and structure-function of beta-toxin.

## 1. Introduction

*Clostridium perfringens* is a gram-positive, rod-shaped bacterium. This organism is an anaerobic microorganism, but not a strictly anaerobic bacteria. *C. perfringens* strains elaborate four major toxins, named alpha-, beta-, epsilon- and iota-toxins, which have lethal, necrotic and cytotoxic activities, among others, and are categorized into five groups (types A to E) [1,2,3,4,5]. *C. perfringens* strains are known to be correlates with a variety of infectious disease: myonecrosis in humans and animals is due to type A strains, type B strains cause lamb dysentery, type C strains are associated with necrotizing enterocolitis (e.g., Darmbrand and Pig-bel), type D strains are correlated with enterotoxemia of sheep, and type E strains are the cause of enterotoxemia in calves and lambs. Individual major toxins have been considered to be an essential pathogeic agent in these diseases. *C. perfringens* type C, which produces alpha- and beta-toxin, causes hemorrhagic serious ulceration or mucous necrosis of the small bowel in humans, swine, and cattle [3,4,5]. Beta-toxin is appreciated to be the aetiological factor in necrotizing enterocolitis caused by type C strains [3,4,5].

Beta-toxin belongs to a β-pore-forming toxin family, which includes *Staphylococcus aureus* alpha-toxin, leukocidin, and gamma-toxin [5,6,7]. Cell lines that are susceptible to this toxin have been found. In addition, knowledge on its biological features, pathogenic role and action mechanism of beta-toxin has also been pooled. This review outlines recent knowledge on this issue and deals with the mechanism of beta-toxin.

## 2. Pathogenesis of *C. perfringens* Type C

Beta-toxin is elaborated by *C. perfringens* type B and C strain isolates and is the essential pathogenic agent of necro-hemorrhagic enteritis induced by *C. perfringnes* type C [4,5,6,7]. *C. perfringens* type C strain isolates also induce lethal infections ranging from necro-hemorrhagic enterocolitis to enterotoxemia in pigs, cattle, sheep and goats, mainly in neonatal animals of numerous domestic animal species, in which the organism propagates in the small bowel and elaborates toxins [4,8,9]. Even though mature animals can contract such illness, they most often occur in the young animals [10]. Piglets are highly sensitive to type C infectious diseases [11,12], although similar infections occur in newborn calves [13], lambs [14] and goats. During periods of a type C infection, necro-hemorrhagic enteritis can be extensive, following incorporation of beta-toxin from the small bowel into the systemic circulation. Neurological symptoms such as tetanic contraction and opisthotonos have been recognized in those animals prior to death [4], suggesting the related neurological symptoms are attributed to toxins that are elaborated in the bowels but then uptaked into the circulation to influence viscera such as the brain. In naturally occuring necro-hemorrhagic enteritis in piglets, beta-toxin was shown to bind to vessel endothelial cells in the enteric mucosa [15,16]. In unvaccinated herds, the mortalities can reach 100%, causing significant economic losses [4,9].

In humans, strains of type C induce food-borne necrotizing enterocolitis (also named as Darmbrand or Pig-bel), which is an endemic disease in the Highland of Papua New Guinea [17,18]. The human-type infection is historically most strongly related to the Highland of Papua New Guinea, where it is recognized as Pig-bel and occurs in individuals after the ingestion of insufficiently cooked pork during certain ritualistic ceremonies [17,19]. Affected individuals with Pig-bel in Papua New Guinea present with serious bloody diarrhea, abdominal pain, distension and emesis. Surgical excision of necrotic tissues of the intestine is the last way to save patients. As Pig-bel in Papua New Guinea results from an increased consumption of pork, it is proposed that the illness is related to the intake of a high-protein food. More specifically, an essential agent in the severity of the disease is thought to be a change from a low-protein food (based on “kaw kaw”, sweet potato containing trypsin inhibitors that contribute to preservation of the protease-labile beta-toxin in Papua New Guinea) to a high-protein food. Low-protein ingestion for long periods generally appears to amount to chronic protein nutritional deficiency in peoples. As such, it is assumed that the circumstance in Germany just after World War II [18] was equal to that in Papua New Guinea. It is suggested that the abovementioned condition may be associated with an enteric canal mainly conditioned to a vegetarian diet unexpectedly being confronted with animal protein-rich diet [20]. Prior to vaccination campaign undertaken during 1970s and 1980s, type *C*-induced necrotizing enterocolitis was the most common etiology for death in children over the age of one in the Highlands of Papua New Guinea [17,18,19,20,21].

Necrotizing enterocolitis caused by type C isolate is marked by mucosal necrosis influencing several regions of the proximal jejunum, which was validated by the endoscopic examinations observed in several cases. Necrotizing enterocolitis associated with type C isolates has been published in some countries [22,23,24,25]. A study of ten sporadic cases (five male, five female) indicated that the mean age was 43.1 years (12 to 66) [25]. Four patients died, showing a high death rate. Six patients had insufficiently controlled or nontreated diabetes, which could be associated with morbidity of infectious diseases, delayed gastric emptying and reduced intestinal mobility. Early manifestations were bloody diarrhea in three cases and abdominal pain in seven cases. Diets that had been taken by the affected individuals were of animal source in three cases, seafood in two cases, turkey or chicken in two cases, food of vegetable source in one case, and none in two cases, indicating that there were no particular associations with particular foodstuffs [25]. The small intestine was influenced in eight cases and the colon in two cases. The infectious disease manifestations varied depending upon the term of illness and its seriousness, but general observations were mucosal necrosis with or without the formation of pseudomembrane, emphysema, and bleeding of the submucosal tissue [25]. Many toxins produced by bacteria were probably related to the mucosal necrosis. The alpha-toxin has phospholipase C and sphingomyelinase activities that destroy cell membranes by cleaving phosphatidylcholine and sphingomyelin. The beta-toxin causes transmural intestinal necrosis [25]. The risk factors for progression to this infectious disease contain decreased production of trypsin responsible for an inadequate protein food or pancreatic disease and the ingestion of diets containing an enrichment of trypsin inhibitor. Individually, these risk factors play a role in persistence of toxin in the alimentary tract during infection of type C. Spontaneous *C. perfringens* type *C*-induced necrotizing enterocolitis in humans showed acute, deep necrosis of the enteric mucosa accompanied with acute necrosis of blood vessel and severe hemorrhage in the submucosa and lamina propria. The disease is also raised in diabetic subjects. Immunohistochemical studies of tissue samples from a diabetic patient who died of necrotizing enterocolitis showed endothelial binding of the toxin in enteric lesion sites [25].

The administration of the toxoid type C filtrates containing of beta-toxin to Papua New Guinea tribes people resulted in a dramatic decrease in the frequency of necrotizing enterocolitis [26,27]. Springer and Selbitz [28] also reported that vaccination of sows with *C. perfringens* type C toxoid-containing vaccine and simultaneous treatment of a penicillin antibiotic preparation in piglets results in a marked decrease in piglet losses. Furthermore, we showed that immunization with the oligomer of the toxin, the inactive conformation, rescued mice from an intraperitoneal admistration with a culture filtrate of type C strains derived from Pig-bel patients [5]. Previous vaccination with an inactivated crude toxin preparation gained from culture filtrates of type C strains also protected against the development of the disease [1]. On the occasion of agricultural domestic livestock, immunization against type C enterocolitis is generally proposed in order to avoid destructive losses [4,20]. Beta-toxin is largely responsible for the mortality of type C infections, but we understand very little about its mode of action or the sensitive cell types. Several groups recently reported recombinant attenuated beta-toxin vaccines. Recombinant beta-toxoid produced by *E. coli* showed the elicitation of protective antitoxin antibody in rabbits [29]. Salvarani *et al.* [30] also reported that *C. perfringens* recombinant alpha-toxoid and beta-toxoid can be regarded as candidates for the establishment of a vaccine against *C. perfringens* type C. Recently, Bhatia *et al.* [31] reported that recombinant B-subunit of *E. coli* heat labile enterotoxin-putative B-cell epitope of beta-toxin (140–156 amino acid residues) fusion protein is a potential vaccine candidate against beta-toxin.

## 3. Evidence for the Involvement of Beta-Toxin

Current experimental investigations employing rabbit intestinal loop model and mice oral or intestinal inoculation models apparently showed that beta-toxin is the important pathogenic agent of type C strains, as described by usage of purified beta-toxin or isogenic null mutants of type C isolates [32,33,34]. As type C isolates typically produce alpha-toxin, beta-toxin and perfringolysin O, the McClane group evaluated the roles of these toxins to the pathogenesis of type C disease using single and double isogenic toxin null mutants of type C disease isolate CN3685 [32,35,36,37,38]. In rabbit intestinal loops, inoculation of wild-type CN3685 induced necrosis of villous tip, which showed that there was early intestinal epithelial injury. On the other hand, the *cpb* null mutant induced neither intestinal necrosis nor accumulation of bloody fluid in rabbit intestinal loops [33]. Additionally, complementing the *cpb* null mutant to recover beta-toxin production notably elevated intestinal pathogenesis. On the other hand, a double mutant of CN3685 that did not produce alpha-toxin or perfringolysin O retained sufficient virulent in rabbit ileal loops. Furthermore, in the presence of trypsin inhibitor, beta-toxin induced bloody fluid accumulation in rabbit ileal loops. In the mouse model, wild-type CN3685 was 100% lethal, yet an isogenic *cpb* null mutant showed largely decreased lethality. In the meantime, an isogenic CN3685 double mutant failed to produce alpha-toxin or perfringolysin O exhibited only a modest reduction in lethality. Gurtner *et al.* [15] showed endothelial localization of beta-toxin in acute lesion sites of spontaneously developing necrotizing enterocolitis in piglets. Furthermore, anti-beta-toxin antibody blocked the intestinal pathogenesis of wild-type CN3685 [32]. Purified beta-toxin produced the intestinal injury of wild-type CN3685, and this damage was protected by anti-beta-toxin antibody [15]. Taking these finding together, the above studies suggest that beta-toxin plays a crucial role in the pathogenesis of type C isolate.

Naturally occurring *C. perfrigens* type C enteritis in piglets showed binding of beta-toxin to vascular endothelial cells in lesion sites of necrotizing enterocolitis, which suggests that beta-toxin could cause vascular necrosis, hemorrhage and then hypoxic necrosis [39]. In a neonatal piglet jejunal loop model, beta-toxin was recognized in microvascular endothelial cells in intestinal villus during initial and development stages of lesion sites caused by *C. perfringens* type C enteritis, indicating that beta-toxin-caused endothelial injury is closely correlated with the initial stage of *C. perfringens* type C infection [40,41]. A direct binding between beta-toxin and endothelial cells might induce vascular necrosis and target destruction of endothelial cells, contributing to the virulence of necrotizing enterocolitis [15,16].

## 4. Beta-Toxin Gene and Regulation of Expression

*C. perfringens* type B and C strain isolates carry the *cpb* gene. The beta-toxin gene exists in large plasmid DNAs in *C. perfringens* that also include the insertion sequence IS1151 [42,43]. Type B isolates contain either 65-kb or 90-kb *cpb*-encoding plasmids [44,45]. Type C strains are known to carry the beta toxin-coding gene on some plasmids ranging from ~65 to ~110 kb [45,46].

Vidal *et al.* [38] reported that many type C strains produce alpha-toxin, beta-toxin and perfringolysin O much more rapidly upon close interact with human intestinal Caco-2 cells than during their *in vitro* growth without these cells. Quick Caco-2 cell-caused up-regulation of the production of beta-toxin participates in the VirS/VirR two-component system, since increased *in vivo* transcription of the *cpb* and *pfoA* genes is inhibited by inactivating the *virR* gene. This is regained upon complementation to recover *virR* expression [35]. Moreover, this two-component system was shown to be needed for type C strain CN3685 to induce the production of beta-toxin *in vivo* and cause either enterotoxemia or necrotizing enteritis in experimental animal models [35]. Therefore, the quick Caco-2 cell-caused toxin production denotes the host cell:pathogen crosstalk influencing production of toxin, which controls VirS/VirR [38,45]. *C. perfringens* contains a chromosomal operon with partly homology to the *S. aureus* operon coding components of the accessory gene regulatory (Agr) quorum-sensing system [45]. A recent study showed that the Agr-like quorum sensing system modulates the elaboration of beta-toxin and is needed for CN3685 to induce necrotizing enterocolitis [36]. In particular, by employing agrB null mutants and their complemented mutants, it was shown that the Agr-like quorum sensing system is necessary for CN3685 to induce intestinal damage in experimental animal models [36]. The dependence of CN3685 pathogenesis on the Agr-like quorum sensing system was demonstrated, in part, to be associated with the system’s regulation of enteric beta-toxin elaboration [36].

## 5. Characterization of Beta-Toxin

Beta-toxin is a 34,861 Da protein, with the gene that encodes it being localized on virulence plasmids in *C. perfringens* [46]. The toxin is dermonecrotic and lethal, but non-hemolytic. Beta-toxin is extremely labile and highly sensitive to thiol group reagents and proteases [5,47,48]. The toxin (monomer) easily changes into the non-toxic oligomer in buffer [5]. Thereby, the beta-toxin-evoked pathogenesis can only occur under specific conditions.

Beta-toxin is remarkably related at the amino acid levels to pore-forming toxins produced by *Staphylococcus aureus*: the A and B components of gamma toxin (22% and 28% similarity), alpha-toxin (28% similarity), and the S and F components of leukocidin (17% and 28% similarity) [5,49]. *S. aureus* alpha-toxin forms heptameric oligomer and inserts itself into cell membranes, leading to formation of functional pore [49]. The alpha-toxin is the archetype of a family member of those toxins with membrane-injuring activity. The above mentioned sequence similarity, thus, suggests that beta-toxin is also a member of pore-forming toxins [49,50,51]. Sakurai *et al.* [47,52] showed that beta-toxin activity is inhibited by sulfhydryl group-reactive reagents. This toxin has one cysteine residue at position 265, but the substitution of Cys-265 with Ser or Ala did not influence its activity [53]. However, the replacement of Cys-265 with a bulky side chain reduced its activity. Furthermore, replacement of residues (Y266, L268 and W275) in the vicinity of Cys-265 resulted in a complete loss of lethal activity [53]. These results suggest that the area near the Cys-265 in the toxin is needed for binding to this toxin’s receptor or formation of oligomer. Although the structure-activity relationship of beta-toxin has remained unclear, an early site-directed mutagenic study reported that the receptor-binding site of the toxin may be present in its *C*-terminal region [51].

## 6. Mechanism of Action of Beta-Toxin

After beta-toxin was administered intravenously into rats, an elevation in blood pressure and a reduction in heart rate were concomitantly recognized [54,55]. Beta-toxin attacks on the autonomic nervous system control and then causes arterial contraction by the liberation of catecholamines, so it elevates the blood pressure. When injected into the skin of mice, the toxin induces dermonecrosis and edema. We previously described that beta-toxin causes the liberation of substance P, an agonist of tachykinin NK_1_ receptor, which is involved in the subsequent neurogenic plasma extravasation [56]. Furthermore, substance P liberated by the beta-toxin from sensory neurons causes the liberation of TNF-α, and these agents are responsible for plasma extravasation [57]. These results indicate that beta-toxin directly or indirectly attacks the central and the peripheral nerves.

The Pothaus group revealed the binding of the toxin to intestinal endothelial cells in naturally infected pigs, a human, and in experimental pig models [39,41]. Furthermore, beta-toxin was shown to be extremely virulent to primary porcine and human endothelial cells and caused acute cell death [15,16]. Hence, it appears that a direct action of beta-toxin on endothelial cells in the bowel plays a role in the virulence of intestinal damage caused by *C. perfringens* type C strains. Beta-toxin was also found to cause necrosis in porcine endothelial cells [58]. Incubation of these cells with beta-toxin resulted in the typical biochemical and morphological behaviors of cells that had died due to necrotic cell death. Beta-toxin-caused necrosis included stimulation of the cell signaling events participating in calpain activation [58].

Steinthorsdottir *et al.* [59] found that beta-toxin formed an oligomeric complex on human umbilical vein endothelial cells. This toxin is known to shift readily to a multimeric form *in vitro*. The Tweten group [60,61] reported that beta-toxin could form potential-dependent, cation-selective channels in planar lipid bilayers composed of phosphatidylcholine and cholesterol (1:1). The pore sizes were determined to be approximately 12 Å in diameter, indicating that the toxin is an oligomerizing, pore-forming protein toxin. Beta-toxin causes swelling and disruption of the human leukemic HL-60 cell line [62]. Given that the incubation of cells with the beta-toxin caused the concurrent incorporation of Ca^2+^, Na^+^ and Cl^−^ into them, and that the toxin-caused Ca^2+^ incorporation and swelling were strikingly inhibited by polyethylene glycols 600 and 1000, it seems likely that the morphological alterations in the cells caused by the beta-toxin is caused via functional pores formed by the toxin in the cell membrane [62]. We reported that beta-toxin formed an oligomeric pore of 228 kDa, which is related to its cytotoxic effect, in lipid raft microdomains of HL-60 cells [62]. Methyl-beta-cyclodextrin, an agent involved in the selective encapsulation of membrane cholesterol, inhibited beta-toxin binding to lipid raft microdomains and cytotoxicity caused by the toxin. Additionally, the treatment of liposomes with beta-toxin induced the oligomer formation of the 228 kDa of the toxin in liposomal membranes and the release of carboxyfluorescein from them. It seems likely that the complex of 228 kDa is a pore, showing that beta-toxin easily forms an oligomer as a functional pore in plasma membranes [62]. From these observations, it is clear that the toxin binds to specific receptors located principally in the lipid raft microdomains of HL-60 cells, forms functional pores in these rafts and causes cytotoxicity. We also reported that beta-toxin induce cell death of five human hematopoietic tumor cell lines (HL-60, U937, THP-1, MOLT-4 and BALL-1) [63]. U937 and THP-1 were highly susceptible to beta-toxin compared with HL-60. We further indicated that the toxin bound preferentially to susceptible cells. We showed that the toxin acts on various immune cells. Potassium efflux by the toxin in THP-1 cells caused the phosphorylation of p38 MAP and JNK kinases. This stimulation was not necessary for the toxin-caused cell death; partly, it was a stress reaction to beta-toxin for survival of cells, indicating that the MAP kinase-signaling cascade plays an important role in the protection from beta-toxin-induced cytotoxicity on THP-1 cells [63].

## 7. Conclusions

Beta-toxin produced by *C. perfringens* types B and C is known as the major virulence agent of necrotizing enterocolitis and enterotoxemia in humans and animals. Beta-toxin formed a functional pore of 228 kDa, which is associated with its cytotoxicity, in the lipid raft microdomains of sensitive cells. Additional study of the mode of action of beta-toxin has provided great insights into the implementation of new preventive strategies and the discovery of novel treatments. Our comprehension of the effect of beta-toxin in virulence of type C infectious disease is restricted, but the identification of its action mechanism should confer a basis for further investigation that may clarify its effect at the molecular and cellular levels and its predominant contribution to virulence. Elucidation of the accurate association of toxins with specific receptor would permit to design specific pharmacological inhibitors or to generate toxin molecules with modified specificity. Some progress has been made in developing vaccines against beta-toxin. Vaccines of varying quality are available to combat type C infection, for animals and humans use. Clearly, there is much more to learn regarding beta-toxin, how it works and how to protect against it.
